# Predictive value of initial FDG-PET features for treatment response and survival in esophageal cancer patients treated with chemo-radiation therapy using a random forest classifier

**DOI:** 10.1371/journal.pone.0173208

**Published:** 2017-03-10

**Authors:** Paul Desbordes, Su Ruan, Romain Modzelewski, Pascal Pineau, Sébastien Vauclin, Pierrick Gouel, Pierre Michel, Frédéric Di Fiore, Pierre Vera, Isabelle Gardin

**Affiliations:** 1 LITIS Quantif – EA4108, University of Rouen, Rouen, France; 2 Dosisoft, Cachan, France; 3 Nuclear Medicine Department, Henri Becquerel Centre, Rouen, France; 4 Normandie Univ, UNIROUEN, Inserm 1245, Rouen University Hospital, Department of Hepato-gastroenterology, Rouen, France; 5 Department of Oncology, Henri Becquerel Centre, Rouen, France; University of South Alabama Mitchell Cancer Institute, UNITED STATES

## Abstract

**Purpose:**

In oncology, texture features extracted from positron emission tomography with 18-fluorodeoxyglucose images (FDG-PET) are of increasing interest for predictive and prognostic studies, leading to several tens of features per tumor. To select the best features, the use of a random forest (RF) classifier was investigated.

**Methods:**

Sixty-five patients with an esophageal cancer treated with a combined chemo-radiation therapy were retrospectively included. All patients underwent a pretreatment whole-body FDG-PET. The patients were followed for 3 years after the end of the treatment. The response assessment was performed 1 month after the end of the therapy. Patients were classified as complete responders and non-complete responders. Sixty-one features were extracted from medical records and PET images. First, Spearman’s analysis was performed to eliminate correlated features. Then, the best predictive and prognostic subsets of features were selected using a RF algorithm. These results were compared to those obtained by a Mann-Whitney U test (predictive study) and a univariate Kaplan-Meier analysis (prognostic study).

**Results:**

Among the 61 initial features, 28 were not correlated. From these 28 features, the best subset of complementary features found using the RF classifier to predict response was composed of 2 features: metabolic tumor volume (MTV) and homogeneity from the co-occurrence matrix. The corresponding predictive value (AUC = 0.836 ± 0.105, Se = 82 ± 9%, Sp = 91 ± 12%) was higher than the best predictive results found using the Mann-Whitney test: busyness from the gray level difference matrix (*P* < 0.0001, AUC = 0.810, Se = 66%, Sp = 88%). The best prognostic subset found using RF was composed of 3 features: MTV and 2 clinical features (WHO status and nutritional risk index) (AUC = 0.822 ± 0.059, Se = 79 ± 9%, Sp = 95 ± 6%), while no feature was significantly prognostic according to the Kaplan-Meier analysis.

**Conclusions:**

The RF classifier can improve predictive and prognostic values compared to the Mann-Whitney U test and the univariate Kaplan-Meier survival analysis when applied to several tens of features in a limited patient database.

## Introduction

In oncology, to diagnose, describe the tumor stage, and monitor the response to therapy, FDG-PET based on the standard uptake value (SUV) is widely used [1]. Predictive and prognostic studies have already been carried out using image features derived from first-order statistics, such as MTV or total lesion glycolysis (TLG). In solid tumors, predictive and prognostic values have been found for these features [2].

More recently, new first-order features have been proposed to describe the heterogeneity of FDG uptake in lesions. For instance, Bundschuh et al. [3] have found that the coefficient of variation (COV) is an important predictive factor in patients with rectal cancer. El Naqa et al. [4] have proposed extracting features from the SUV-volume histogram (SVH), such as SUV_x_ (the minimum SUV of the x% highest SUV) and V_x_ (the percentage of volume having at least x% of SUV). These authors have found that features extracted from the gray-level co-occurrence matrix (GLC matrix) [5] characterizing the intensity relationships between pairs of neighboring voxels are some of the most important predictive features in cervical cancer. Other texture matrices have also been proposed in the literature. The gray-level difference matrix (GLD matrix) [6] characterizes the intensity differences between neighbors and the gray-level run length (GLRL matrix) [7] and the gray-level size zone (GLSZ matrix) matrices [8], which characterize the range of intensities in a direction or in all directions, respectively. All these features, called radiomics features, provide great potential to capture important phenotypic information, such as intra-tumor heterogeneity and valuable information for personalized therapy [9]. Nevertheless, one challenge is the establishment of a proper study design to manage several tens of characteristics per lesion.

Several studies investigating the prognostic and predictive value of initial FDG-PET features in patients with esophageal cancer treated by chemo-radiation therapy (CRT) have been proposed in the literature [10]–[17]. When MTV is studied, it always appears to be predictive and prognostic. Moreover, Tixier et al. [17] have found that features derived from GLC, GLD, and GLSZ matrices are predictive of a complete response (CR).

Because of the high number of studied features and the nonlinear pattern relationships between features and patient outcome, the mathematical tools used in these studies are not sufficiently powerful. In this context, methods based on machine learning could lead to a better discriminant power than classical statistics when analyzing several tens of features. These methods are able to learn from data by selecting a subset of complementary features leading to the prediction of patient outcome [18]. Several algorithms have been proposed in the literature for radiomics applications in computed tomography (CT) [18] [19]. Among them, methods based on RF algorithms provide promising results.

Many radiomics features can be extracted from data, but they do not necessarily improve the accuracy of the prediction due to information redundancy. Some are correlated [20] [21]. For instance, Orlhac et al. [22] showed that some texture features are highly correlated with MTV in 3 types of tumors: metastatic colorectal cancer, non-small cell lung cancer, and breast cancer. Tixier et al. [17] have shown that GLRL matrix features are highly correlated with GLSZ matrix features, and, therefore, do not provide complementary information.

In this study, in order to predict treatment response and patient survival based on baseline FDG-PET images in a database of 65 patients with locally advanced esophageal cancer after CRT using 61 features extracted per patient. This method was compared to another feature selection method based on a support vector machine (SVM) as well as to a standard statistical analysis: the Mann-Whitney U test for predictive study and the univariate Kaplan-Meier analysis for prognostic study.

## Materials and methods

### Patient population

Sixty-five patients with 1 lesion histologically proven to be locally advanced esophageal cancer were included in the study. All procedures performed in this study were conducted according to the principles expressed in the Declaration of Helsinki. The study was approved as a retrospective study by the Henri Becquerel Center Institutional Review Board (number 1506B). All patient information was de-identified and anonymized prior to analysis. From the clinical and biological data, 16 features were extracted for each patient and integrated into this study (Table 1).

**Table 1:** List of patient features.

Features	Number of patients
*Demographic*	
Patient’s age (years)	
Median (range)	63 (46-85)
Patient’s gender	
Male	54 (83%)
Female	11 (17%)
*Clinical*	
Tumor location	
Upper third	18 (28%)
Middle third	26 (40%)
Lower third	21 (32%)
Histology	
Adenocarcinoma (ADC)	8 (12%)
Squamous cell carcinoma (SCC)	57 (88%)
Clinical Stage	
II	17 (26%)
III	39 (60%)
IV	9 (14%)
*Outcomes*	
3-year survival	
Alive	24 (37%)
Dead	41 (63%)
1-month response	
Complete (CR)	41 (63%)
Non-complete (NCR)	24 (37%)
Follow-up (month)	
Median (range)	23 (6-79)

Patients underwent FDG-PET with a CT before treatment, at the initial stage, and after treatment during systematic follow-up (at 1 month and 3 years) or in cases of clinically suspected recurrence (38/65 patients), always at the same institute. They were treated by CRT between 2006 and 2013 according to the Herskovic scheme [23], including uninterrupted radiation therapy in the form of external radiation delivered by a 2-field technique of 2 Gy per fraction per day, 5 sessions per week, for a total of 50 Gy, as well as chemotherapy including platinum and 5-fluorouracil. The initial tumor staging and location was based on an esophagoscopy with chest and abdominal CT with contrast, endoscopic ultrasonography, FDG-PET/CT, and biopsies. After CRT, 14 patients underwent surgery (4 stage II, 8 stage III, and 2 stage IV).

For the prediction of treatment response, the response assessment included clinical examination, CT, FDG-PET, and esophagoscopy with biopsies performed 1 month after the end of treatment. Patients were classified as showing a clinically complete response (CR, 41 patients) to CRT if no residual tumor was detected on the endoscopy (negative biopsies) and if no locoregional or distant disease were identified on CT or via PET evaluation. Of the 41 patients, 24 were alive at the end of their follow-up. Patients were classified as showing a non-complete response (NCR, 24 patients) if a residual tumor or locoregional or distant disease was detected or if death occurred. None of the patients were alive 3 years after treatment.

The mean follow-up of the total studied population was 27.6 ± 18 months. The overall survival (OS) used for the prognostic study was estimated at 3 years after the end of the CRT. At the end of the follow-up, 24 patients were alive and 41 were dead.

### FDG-PET/CT imaging

The FDG-PET/CT data were acquired on a Biograph^®^ Sensation 16 Hi-Rez device (Siemens Medical Solutions, IL, USA). Patients were required to fast for at least 6 hours before imaging. A total of 5 MBq/kg of FDG was injected after 20 min of rest. Sixty minutes later (±10 min), 6 to 8 bed positions per patient were acquired using a whole-body protocol (3 min per bed position). The PET images were reconstructed using Fourier rebinding and attenuation-weighted ordered subset expectation maximization algorithms. The images were corrected for random coincidences, scatter, and attenuation. Finally, the FDG-PET images were smoothed with a Gaussian filter (full width at half maximum = 5 mm). The reconstructed image voxel size was 4 × 4 × 2 mm^3^.

### Feature extraction

**Fig 1:**
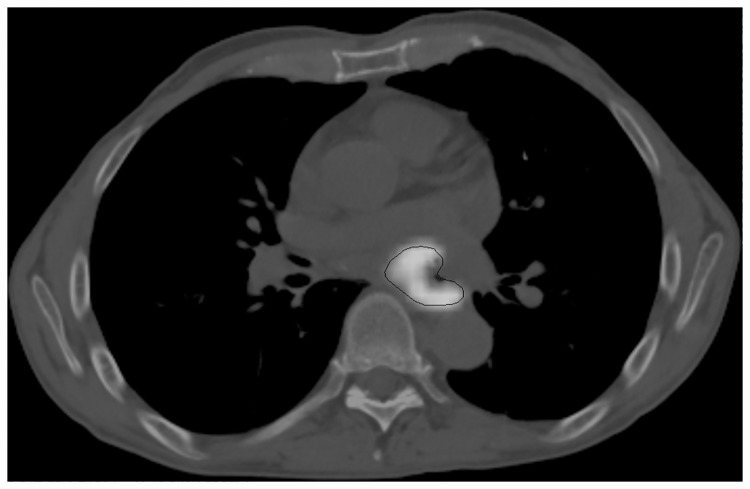
PET/CT slice of the chest with a segmented esophageal tumor.

Forty-five features were extracted from PET images (see Table 2) according to the following workflow. First, MTV was defined using a contrast-based adaptive threshold algorithm [24] on a PLANET Onco workstation (DOSIsoft, Cachan, France). With this tool, it is possible to select all the volume and avoid empty parts in the final segmentation corresponding to necrotic tissues. An example of a FDG-PET/CT chest slice and the segmentation of the lesion are shown in Fig 1. Nineteen 1^st^ order features were extracted based on SUV, MTV, TLG, COV, SVH, and sphericity [25].

**Table 2:** List of the initial features.

Type of features	Features
Clinical data	Patient’s age, Patient’s gender,
	Albumin level (g/l), nutritional risk index (NRI), Malnutrition*,
	Patient’s current weight (kg), Usual weight (kg), Weight loss (%),
	Tumor location (up, mid, low), Histology (ADC or SCC),
	TNM stage, WHO performance status,
	Endoscopic tumor length (cm)
1^st^ order statistics	SUV_max_, SUV_mean_, SUV_peak_, Sum of SUVs (SUV_sum_)
	MTV, TLG, Standard Deviation (SD), COV, Sphericity,
	Skewness, Kurtosis, Energy, Entropy,
	SUV_10_, SUV_90_, SUV_10_-SUV_90_, V_10_, V_90_, V_10_-V_90_
Texture indices**	*GLCM* [5]: Variance, Energy, Entropy, Correlation, Dissimilarity,
	Contrast, Homogeneity, Inverse Differential Moment (IDM),
	Cluster Shadey, Cluster Tendency
	*GLSZM* [8]: Short Zone Emphasis (SZE), Long Zone Emphasis (LZE),
	Low Gray level Zone Emphasis (LGZE), High Gray-level Zone
	Emphasis (HGZE), Short Zone Low Gray-level Emphasis (SZLGE),
	Long Zone Low Gray-level Emphasis (LZLGE), Short Zone High
	Gray-level Emphasis (SZHGE), Long Zone HighGray-level
	Emphasis (LZHGE), Zone Percentage (ZP), Gray Level Non
	Uniformity (GLNUz), Zone Length Non Uniformity (ZLNU)
	*GDLM* [6]: Coarseness, Contrast, Busyness, Complexity, Strength

*absent if NRI > 97.5, average if 83.5 ≤ NRI ≤ 97.5 and severe if NRI < 83.5.

**mathematical expressions of features come from Table 1 of the Supplemental Data from [22]

Second, 26 texture indexes were extracted from 3 texture matrices leading to 10 features for GLC matrix, 5 for GLD matrix, and 11 for GLSZ matrix. To compute these matrices, an absolute linear gray-level resampling was applied on MTV voxels according to [26] and [27]:

$$\begin{array}{c} R_{\text{abs}}\left(i\right)=\text{round}\left(D\times SUV\left(i\right)\right) \end{array}$$ (1)

where *SUV*(*i*) is the initial SUV of voxel *i* and *R*_abs_(*i*) is the new intensity after the absolute resampling process based on *D* the intensity step, set to 0.5.

To compute the GLC matrix, 13 matrices were used, 1 for each spatial direction. Then the matrices were averaged into 1 mean matrix [15]. The mathematical expression of the texture indexes can be found in [22] for the GLC, GLSZ, and GLD matrices, leading to *F*_*i*_ = 61 initial features (Table 2).

### Proposed feature selection strategy based on RF

**Fig 2:**
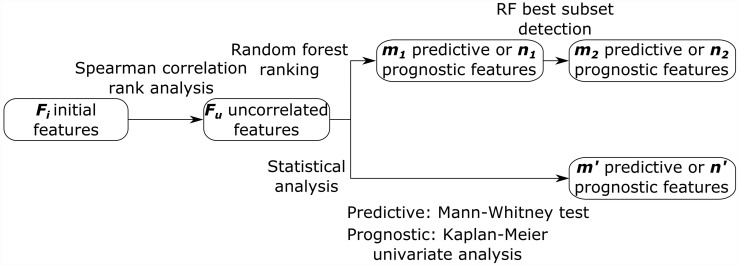
Workflow of the feature selection strategy and data analysis.

The workflow of the feature selection strategy is given Fig 2. To determine predictive and prognostic features, the 61 features were pre-selected to maintain *F*_*u*_ uncorrelated features. Second, a feature selection was performed using the RF algorithm [28] to maintain the most important predictive (and prognostic) features. Then, the subset of complementary features with the best predictive (and prognostic) value was found using RF again.

For the pre-selection step, a study based on a Spearman’s rank correlation analysis was performed with the Statistics Toolbox of MATLAB (version 2014b, MathWorks, Inc., Natick, MA, USA) to maintain the uncorrelated features. Features were compared one by one and considered as significantly correlated if the absolute value of the Spearman’s correlation coefficient (|*ρ*|) was higher or equal to 0.8 with a *P*-value (*P*) smaller than 5% [22]. Correlated features satisfying these conditions were placed in the same group. In each group, the representative feature was the one corresponding to the most robust with respect to image reconstruction settings [29].

After this pre-selection, the most relevant features among those remaining were defined using the RF algorithm. Five hundred decision trees were built leading to the creation of a RF classifier. This classifier was created using the *F*_*u*_ uncorrelated features from the 65 patients. Features were then ranked according to the RF importance coefficient. *m*_1_ predictive and *n*_1_ prognostic features were selected if their coefficient of importance was higher than 10% of the highest one. Second, in order to define the best subset of complementary features among the *m*_1_ (and *n*_1_) remaining, the construction of several RFs was recursively performed based on different subsets of features. Each subset was evaluated using an out-of-bag error. The best subset was the one minimizing this error, ultimately leading to the selection of *m*_2_ predictive and *n*_2_ prognostic features.

### Evaluation and comparisons with other methods

To evaluate our proposed feature selection strategy, a new classification was made based on the training sample reduced to the *m*_2_ predictive (or the *n*_2_ prognostic) features. This evaluation was done with 2 types of classifiers, a RF and a SVM classifier. These classifications were evaluated by comparing ground truth and estimated labels leading to 2 misclassification rates (RF_err_ and SVM_err_). Because of the small number of observations in the database, a validation protocol called random permutations was used. This process randomly divides the database into 2 subsets: two-thirds of the data are used for the training sample and one-third for the test sample. This process is repeated 10 times, leading to an average and a standard deviation of performance indices.

To evaluate the interest of this feature selection strategy, results were compared with other methods. First, the classification was made without any feature selection strategy based on the *F*_*i*_ initial features. To evaluate the contribution of each step of the proposed selection strategy, the classification was made using only the pre-selection step (Spearman’s rank correlation analysis) or only the other step (RF importance coefficients). Finally, results obtained by the proposed method were compared to those obtained by another feature selection strategy based on SVM that is called the hierarchical forward selection method (HFS) [30]. This method led to *m*_HFS_ predictive and *n*_HFS_ prognostic features. These subsets were evaluated in the same way as the proposed method.

### Statistical analysis

The workflow of the statistical analysis, calculated using MedCalc software for Windows (version 12.7, MedCalc Software, Ostend, Belgium), is shown Fig 2.

First, features selected using RF (*m*_2_ or *n*_2_) were combined using a RF in order to turn the subset into 1 feature. The performances of the RF methodology were studied using a receiver operation characteristic (ROC) methodology [31] leading to an area under the curve (AUC), a sensitivity (Se) and a specificity (Sp). Furthermore, for the prognostic study, a Kaplan-Meier analysis was performed leading to median survival, percentage of deaths in each group, and hazard ratio (HR).

Our RF feature selection strategy was compared to a Mann-Whitney U test (predictive study) and to a univariate Kaplan-Meier analysis (prognostic study). This comparison was done on the *F*_*u*_ uncorrelated features (see Fig 2). For the predictive features, relationships between the response to therapy at 1 month and the features were studied using the Mann-Whitney U test. A P value less than 5% was considered to be statistically significant, leading to *m*′ predictive features. ROC methodology was used to assess feature performances 1 by 1 in order to differentiate patients (CR and NCR). To assess the prognostic value of features, a Kaplan-Meier test was used to estimate survival distribution. OS was calculated from the date of initial diagnosis to the date of death or to the end of the follow-up period. The association between OS and each feature was performed after a dichotomization process. The most discriminating cut-off value allowing for the differentiation of the 2 groups of patients was selected using ROC methodology. The prognostic value of each feature in terms of OS was assessed using the log-rank test leading to *n*′ prognostic features. To avoid false conclusions, appropriate statistical corrections for the type-I errors were done according to Chalkidou et al. [32]. For each *P*-value calculated in both predictive and prognostic studies, a Benjamini-Hochberg correction for multiple hypotheses testing was applied [33]. Furthermore, for the prognostic study, a correction of the minimal P values obtained from the optimum cut-off approach was performed using the Altman formula [34].

A Wilcoxon signed-rank test was performed to study whether the methods were statistically different, with an alpha risk of 5% (*P* < 0.05) [35].

## Results

From our database, the mean MTV was 19.6 ± 20.5 cm^3^ (range 2.5-141 cm^3^) and the mean SUV_max_ was 12.3 ± 4.9 (range 3.5-25.6).

Table 3 shows the results of the influence of the different steps of our feature selection strategy. Even if our proposed method gives the best performances for the predictive study, there was no statistically significant difference between the methods, while for the prognostic study, our proposed method was statistically different than the 3 other feature selection strategies (*P* < 0.05).

**Table 3:** : Results of classifications performed without any feature selection, with the proposed method, using only the pre-selection step (Spearman’s correlation analysis) or only the selection by RF algorithm (RF importance coefficients).

Study	Feature selection	RF_err_ (%)	AUC_RF_	Se (%)	Sp (%)
Predictive	Without	25±7	0.798±0.084	74±10	88±12
	Only pre-selection	28±5	0.788±0.074	76±7	85±11
	Only selection by RF	30±8	0.745±0.092	62±16	90±14
	Proposed method	21±9	0.836±0.105	82±9	91±12
Prognostic	Without	34±6	0.677±0.097	78±10	65±22
	Only pre-selection	31±10	0.698±0.085	80±15	68±12
	Only selection by RF	32±11	0.661±0.135	89±13	54±20
	Proposed method	28±5	0.822±0.059	79±9	95±6

Results of the Spearman’s analysis of the 61 initial features are given in Table 4. Concerning clinical data, the patient’s usual weight and current weight were correlated (|*ρ*| > 0.96), as well as the albumin level, the nutritional risk index (NRI) and malnutrition (|*ρ*| > 0.88). This is due to the fact that NRI and malnutrition features were obtained from the albumin level. None of the clinical data were correlated with the other studied features.

**Table 4:** Groups of correlated features (clinical, 1^st^ order and texture) created with an absolute threshold value of the Spearman’s correlation coefficient of 0.8. The feature selected to represent each group for the next step is in bold.

Group	Correlated features
1	**Patient’s usual weight**—Patient’s current weight
2	**NRI**—Albumin level—Malnutrition
3	**V_10_-V_90_**—V_90_
4	**ZLNU**—Cluster Shade (GLCM)
5	**Energy (1^st^ order)**—Entropy (1^st^ order)
6	**MTV**—_sum_SUV—TLG—Correlation (GLCM)
7	**SUV_max_**—SUV_10_—SUV_peak_—SUV_mean_—SD—SUV_10_-SUV_90_—Variance (GLCM)—Cluster Tendency (GLCM)—HGZE (GLSZM)—LGZE (GLSZM)—Complexity (GLDM)
8	**Homogeneity (GLCM)**—IDM (GLCM)—Dissimilarity (GLCM)—Energy (GLCM)—Entropy (GLCM)—Contrast (GLCM)—LZE (GLSZM)—ZP (GLSZM)—LZLGE (GLSZM)—LZHGE (GLSZM)–Contrast (GLDM)—Strength (GLDM)
9	**Busyness (GLDM)**—Coarseness (GLDM)—Sphericity

Only 8 PET image features were not correlated: V_10_, SUV_80_, COV, skewness, kurtosis, SZE, SZLGE, and GLNUz. At the end of this correlation study, 9 groups of significantly correlated features (|*ρ*| ≥ 0.8, *P* < 0.05) were identified. The patient’s weight, NRI, V_10_-V_90_, energy (1^st^ order), MTV, SUV_max_, homogeneity (GLC matrix), busyness (GLD matrix), and ZLNU (GLSZ matrix) were used as leaders of their correlation groups. This step led to the pre-selection of *F*_*u*_/*F*_*i*_ = 28/61 features (13 clinical and 15 from images). Then, classifications using the proposed feature selection strategy were realized based on these uncorrelated features.

Concerning the prediction of the treatment response, the number of the most important predictive features found using the coefficient of importance of RF was *m*_1_ = 9. Table 5 shows the ranking and the corresponding coefficient of importance of these features. At the end of the selection strategy, the best predictive performance was obtained with the following *m*_2_ = 2 complementary features: MTV (group 6) and homogeneity (GLC matrix, group 8), leading to an AUC of 0.836±0.105, a RF misclassification rate of 21±9%, a SVM misclassification rate of 35±6%, a sensitivity of 82±9%, and a specificity of 91±12% (see Table 6). From the Mann-Whitney U test performed on *F*_*u*_ = 28 features, only *m*’ = 5 features had a significant *P*-value (*P* < 0.05): the patient’s weight loss, MTV (group 6), energy (1^st^ order), busyness (GLD matrix) and GLNUz (GLSZ matrix). Table 6 shows the results extracted from the ROC curve analysis of the corresponding significantly predictive continuous features. The highest AUC value (0.810) was obtained with busyness (GLDM, group 9). This value is lower than the one found using RF. The best predictive performances of HFS were obtained with *m*_HFS_ = 4 features. Even if our method gives better performances than HFS, there was no statistically significant difference between the 2 methods for the predictive study.

**Table 5:** Ranking of the most important predictive and prognostic features according to the value of the coefficient of importance (CI) calculated by the RF algorithm. Correlation group is indicated if necessary. In bold, the features selected during the last step of selection leading to the best subsets of features.

Rank	Predictive features	CI	Prognostic features	CI
	*m*_1_ = 9 and *m*_2_ = 2		*n*_1_ = 8 and *n*_2_ = 3	
1	**MTV (group 6**)	0.534	**NRI (group 2)**	0.272
2	GLNUz	0.319	Patient’s age	0.257
3	Busyness (GLDM, group 9)	0.236	**WHO performance status**	0.200
4	Energy (1^st^ order, group 5)	0.220	Patient’s weight loss	0.155
5	**Homogeneity (GLCM, group 8)**	0.181	**MTV (group 6)**	0.149
6	Patient’s weight loss	0.166	Tumor location	0.089
7	Patient’s usual weight (group 1)	0.128	SZE (GLSZM)	0.081
8	WHO performance status	0.114	Energy (1^st^ order, group 5)	0.077
9	Contrast (GLCM)	0.071	-	-

**Table 6:** Results of the prediction of treatment response using the proposed method based on RF, the HFS method or the Mann-Whitney U test (*p* < 0.05). ROC curves were created to obtain sensitivity (Se), specificity (Sp) and AUC.

Features	Se	Sp	AUC	RF_err_ (%)	SVM_err_	Mann-Whitney test
**Proposed method (*m*_2_ = 2)**						
Subset of complementary features:	82±9	91±12	0.836±0.105	21±9	35±6	-
- MTV (group 6)						
- Homogeneity (GLCM, group 8)						
**HFS (*m*_HFS_ = 4)**						
Subset of complementary features:	77±15	86±15	0.814±0.093	29±12	37±8	-
- Patient’s weight loss						
- MTV (group 6)						
- Homogeneity (GLCM, group 8)						
- Energy (1^st^ order, group 5)						
- ZLNU (GLSZM, group 4)						
**Mann-Whitney U test (*m*’ = 5)**						
Busyness (GLDM, group 9)	66	88	0.810	-	-	< 0.0001
MTV (group 6)	51	100	0.802	-	-	0.0001
Patient’s weight loss	61	83	0.737	-	-	0.0015
Energy (1^st^ order, group 5)	54	88	0.723	-	-	0.0030
GLNUz (GLSZM)	76	75	0.718	-	-	0.0037

**Fig 3:**
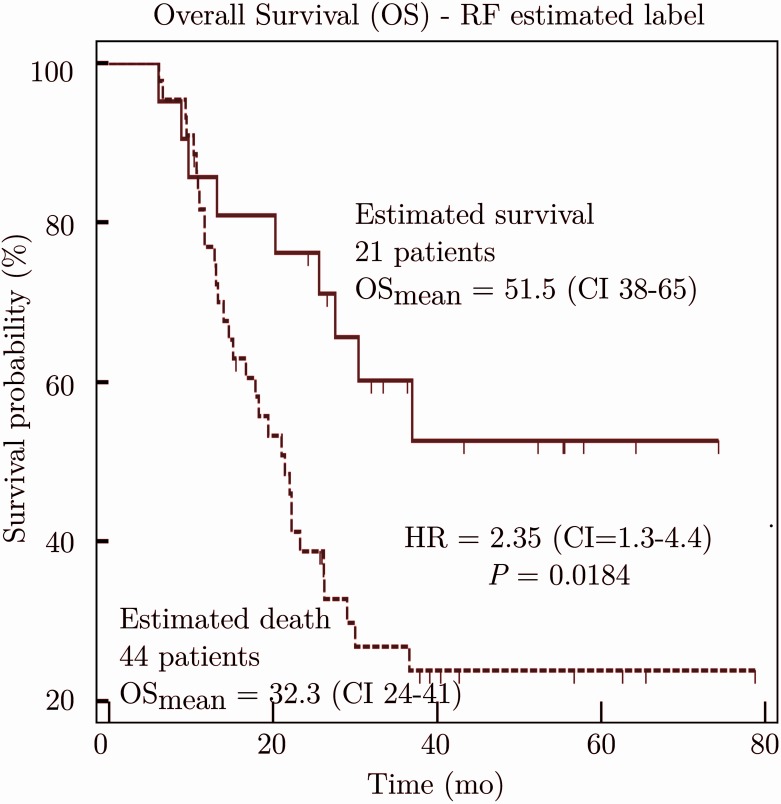
Kaplan-Meier survival curves using the random forest classifier with the best prognostic subset of features defined by the proposed method.

Concerning the prediction of survival, the number of the most important prognostic features found with the RF selection strategy was *n*_1_ = 8 (Table 5). At the end of the selection strategy, the best prognostic performances were obtained with *n*_2_ = 3 complementary features: NRI (group 2), WHO performance status, and MTV (group 6), which led to an AUC of 0.822±0.059 (RF_err_ = 28±5%, SVM_err_ = 34±5%, Se = 79±9%, Sp = 95±6%; see Table 7). Fig 3 shows the Kaplan-Meier survival curves based on estimated labels using the RF classifier and the *n*_2_ features. The corresponding HR was 2.35. The best prognostic performances of HFS were obtained with 3 different features. Our proposed method was statistically different than HFS (*P* = 0.002). Conversely, no feature was detected as significantly prognostic using the Kaplan-Meier survival analysis.

**Table 7:** Prognostic results of the different features using the proposed method based on RF, the HFS method or the univariate Kaplan-Meier analysis (*p* < 0.05). ROC curves were created to obtain sensitivity (Se), specificity (Sp) and AUC.

Features	Se (%)	Sp (%)	AUC	RF_err_ (%)	SVM_err_
**Proposed method (*n*_2_ = 3)**					
Subset of complementary features:	79±9	95±6	0.822±0.059	28±5	34±5
- NRI (group 2)					
- WHO performance status					
- MTV (group 6)					
**HFS (*n*_HFS_ = 3)**					
Subset of complementary features:	55±26	74±19	0.561±0.090	45±8	49±8
- T stage					
- N stage					
- Energy (group 5)					
**Univariate Kaplan-Meier Analysis (*n*’ = 0)**					
None	-	-	-	-	-

## Discussion

On the basis of a cohort of 65 patients suffering from esophageal cancer and treated by CRT, our study shows that an accurate predictive and prognostic value can be found using a RF algorithm. For the predictive study, results obtained with the proposed method (see Tables 3 and 6) are better than the standard statistics (Mann-Whitney U test and a ROC analysis) and are not significantly different than those found with the HFS algorithm. The prognostic study (Tables 3 and 7 and Fig 3) also indicates the good performance of the algorithm. The results are significantly better than those obtained with HFS (*P* = 0.002) and the Kaplan-Meier survival analysis (no feature was detected as significantly prognostic).

The predictive and the prognostic value of radiomics features concerning esophageal cancer have been studied in the literature in FDG-PET [9] [17] [36]–[40], CT [41] [42] and magnetic resonance imaging (MRI) (apparent diffusion coefficient from diffusion weighted imaging) [43]. In other pathologies, some studies have shown that combining 2 imaging modalities can improve the prediction of radiomics features [44]–[47]. Several authors have also proposed studying the temporal features extracted from initial and post-treatment FDG-PET images [9] [36]–[40].

To obtain prognosis and predictive information as soon as possible, most research has focused only on initial FDG-PET features. A few studies have also investigated this issue. Giganti et al. [43] have studied the relationship between apparent diffusion coefficient extracted from MR images and OS in esophageal cancer. In a univariate analysis, this feature had a prognostic value both on the total population (*P* = 0.016) and the surgery-only group (*P* < 0.001). Tixier et al. [17] have studied the prediction of response to CRT in esophageal cancer of 38 textural features from pretreatment FDG-PET images on 41 patients using a Kruskal-Wallis non-parametric test. They found that tumor textural analysis can provide non-responder, partial-responder, and complete-responder patient identification with higher sensitivity (76%-92%) than any SUV measurement. Ganeshan et al. [41] studied the prognostic value of CT features in 21 patients. The authors extracted a large number of features from CT images modified by different Laplacian of Gaussian spatial band-pass filters. Based on a Kaplan-Meier survival analysis, uniformity at a coarse filter scale value of 2.5 led to a significantly prognostic AUC (AUC = 0.769, *P* = 0.0112). Kaplan-Meier curves obtained with this feature were significantly better than with SUV or clinical stage (*P* = 0.0006 vs *P* = 0.0032, *P* = 0.023, respectively). Finally, a Cox regression analysis showed that coarse uniformity was an independent prognostic feature (*P* = 0.039), while the clinical stage was not significant. Giganti et al. [42] also investigated the association between preoperative texture analysis from multidetector CT and OS in gastric cancer. Given the number of features (107) and the number of patients (56), a feature selection was done based on a random survival forest. This study showed that multidetector CT texture analysis is a promising, non-invasive diagnostic tool to evaluate the aggressiveness of gastric cancer.

Given the limited number of patients in our database (65), only 45 FDG-PET features were extracted, while many other features have been proposed in the literature. The chosen features correspond to those generally studied in the literature [17] [22] [36]. Nevertheless, a larger patient cohort will help to integrate more FDG-PET, CT, or MRI features. It should also be noted that our database is composed predominantly of SCC patients (88%). This ratio is of the same order of magnitude as the database used by Tan et al. [36]. Nevertheless, results can depend on the initial patient database.

Several methods have been proposed in the literature to reduce the number of features by eliminating those with an insufficient repeatability and reproducibility [26] [48] [49]. This strategy is included in the pre-selection step of our feature selection, because for each group, the representative feature was the one corresponding to the most robust with respect to image reconstruction settings [29].

Even if pre-selection is performed, the number of uncorrelated features remains high. In general, a nonlinear relationship exists between a feature and the patient outcome. Furthermore, complementary features can improve the prediction. In this context, methods using machine learning-based classifiers are mandatory. Among the algorithms proposed in the literature for radiomic applications, RF has given promising results [18, 19]. To evaluate the performances of our RF algorithm, a comparison was done with a feature selection strategy (HFS) based on SVM. The results are significantly better with our method than with those obtained with HFS (*P* = 0.002 for the prognostic study and was not significant for the predictive study). To avoid bias due to the use of the same classifier between the feature selection strategy and the final evaluation of the method, both our feature selection method and HFS were evaluated using RF and SVM. Here again, regardless of the classifier used for the evaluation, the best results were found with our method (see Tables 6 and 7).

For all statistical approaches, when using machine learning, it is better to have a large database. The principle is to perform a partition of training and evaluation data. Unfortunately, this is generally difficult to obtain from clinical studies. As a surrogate, the same database for both the training and evaluation process was used [50]. As the number of patients in the database (65) is only 2.32-fold *F*_*u*_, a random permutation of 10 iterations was used to avoid overfitting.

Our proposed selection strategy consists of 2 successive selections, reducing the number of features used to build the classification model. The first is a Spearman’s rank correlation analysis, which only keeps uncorrelated features for the next step (*F*_*u*_ = 28/61). The second is based on the RF coefficient of importance. The combination of these 2 steps improves the classification performances with respect to the use of only 1 (Table 3).

We have already developed a two steps combination for features selection [51] using a genetic algorithm for the second step rather than a method based on the coefficient of importance. This method, called GARF (genetic algorithm based on random forest), was evaluated on the same database. AUCs were improved using the coefficient of importance with a smaller number of selected features, if compared to the genetic algorithm (2 in the predictive and 3 in the prognosis studies against 9 in the predictive and 8 in the prognostic studies for GARF). Furthermore, GARF results are sensitive to several parameters of the fitness function of the genetic algorithm leading to optimize these parameters.

During the pre-selection step of our feature selection strategy, a |*ρ*| threshold value of 0.8 was chosen. Two other threshold values (0.7 and 0.9) were also studied (see Table D in S1 File and S1 Fig) without showing a significant difference according to the Wilcoxon signed-rank test. Thus, a value of 0.8 was used as proposed by Orlhac et al. [22]. Most of the correlated features in Table 4 are similar to those in [22], but there are differences that can be explained by the resampling equation used. In [22], a relative resampling equation was used, while we have used an absolute resampling method as proposed in the literature [26] [52]. Most relative resampling-based features are highly correlated with MTV for small tumors (less than 10 cm^3^) [15], while this correlation is reduced for absolute resampling-based features, but introducing a strong dependency on SUV_max_. A comparison of these 2 resampling approaches has been done (see Table C in S1 File) showing a statistically significant difference in favor of absolute resampling on our database (*P* = 0.04 in the predictive study and *P* = 0.01 in the prognostic study).

For the construction of the RF, a value of *T* = 500 decision trees was chosen. Other values of *T* were also studied (see S2 Fig) without showing a significant difference using the Wilcoxon signed-rank test. However, it is known that increasing the number of trees does not reduce the classification performance but tends to converge toward good results [28]. According to our experiments, a value of 500 is a good compromise between the performance of the classifier and the computation duration.

Even if the initial database is the same, the best complementary features selected by the RF algorithm may be different from those found for individual features using standard statistics. With RF, patient classification is performed using a subset of *m*_2_ = 2 complementary predictive features and *n*_2_ = 3 prognostic features.

MTV (group 6, Table 6) appears as a relevant feature on both RF and Mann-Whitney U test predictive studies. This result was also found in the literature in the case of esophageal cancer treated by CRT [11]–[13]. The coefficient of importance of MTV computed by the RF algorithm is the highest (see Table 5), highlighting the relevance of this feature. The corresponding AUC of the Mann-Whitney U test (0.802) is smaller than the one found using a set of complementary features selected by RF (AUC = 0.836, MTV, and homogeneity). This last feature is representative of FDG uptake heterogeneity, pointing out the relevance of heterogeneity analysis. Homogeneity represents group 8 in which many texture features are linked (see Table 4).

The prognostic subset of features is composed of 1 image and 2 clinical features (the patient’s age, the WHO performance status, and MTV), leading to a misclassification rate of 28% and HR of 2.35 (see Fig 3). MTV [10] [14] [16] was already presented as prognostic in the literature. On the other hand, no additional image features improved the accuracy of the patient classification. MTV corresponds to the first prognostic image feature in the ranking list determined by the coefficient of importance from RF, but only in 5^th^ positions in this ranking list (see Table 5).

## Conclusion

Because of the large number of studied features with respect to the number of patients, a RF algorithm was used to determine the subset of complementary features with the highest predictive and prognostic values. We have shown that the RF classifier can improve the predictive and prognostic values compared to the Mann-Whitney U test and the univariate Kaplan-Meier analysis when applied to several tens of features in a limited patient database. Machine learning algorithms are promising for the prediction of treatment response and survival when using several tens of features. Their impact on medical imaging research and clinical routines still has to be evaluated.

## Supporting information

S1 File Mean, standard diviation (SD), median, 1^st^ and 3^rd^ quartile (Q1, Q3) of absolute PET texture features (Table A). Parameters of the RF (Table B). Results of RF classification obtained with two different resampling methods (Table C). Groups of correlated features created with an absolute threshold value of the Spearman’s correlation coefficient varying from 0.7 to 0.9. The feature selected to represent each group for the next step is in bold (Table D).
(PDF)

S1 Fig Results of the RF classification according to the absolute threshold value of the Spearman’s correlation coefficient (a) for the predictive study and (b) for the prognostic study. (TIF)

S2 Fig Results of the RF classification according to *T* the number of trees of the RF (a) for the predictive study and (b) for the prognostic study. (TIF)

## References

[1] Czernin J, Allen-Auerbach M, Schelbert HR. Improvements in Cancer Staging with PET/CT: Literature-Based Evidence as of September 2006. J Nucl Med. 2007;48: 78S–88S. 17204723

[2] Van De Wiele C, Kruse V, Smeets P, Sathekge M, Maes A. Predictive and Prognostic Value of Metabolic Tumour Volume and Total Lesion Glycolysis in Solid Tumours. Eur J Nucl Med Mol Imaging. 2013;40: 290–301. 10.1007/s00259-012-2280-z 23151913

[3] Bundschuh RA, Dinges J, Neumann L, Seyfried M, Zsótér N, Papp L, et al Textural Parameters of Tumor Heterogeneity in 18F-FDG PET/CT for Therapy Response Assessment and Prognosis in Patients with Locally Advanced Rectal Cancer. J Nucl Med. 2014;55: 891–897. 10.2967/jnumed.113.127340 24752672

[4] El Naqa I, Grisby PW, Apte A, Kidd E, Donnelly E, Khullar D, et al Exploring feature-based approaches in PET images for predicting cancer treatment outcomes. Pattern Recogn. 2009;42: 1162–1171. 10.1016/j.patcog.2008.08.011 20161266 PMC2701316

[5] Haralick RM, Shanmugam K and Dinstein I. Textural Features for Image Classification. IEEE T Syst Man Cyb. 1973;SMC-3(6): 610–621. 10.1109/TSMC.1973.4309314

[6] Amadasun M, King R. Textural features corresponding to textural properties. IEEE T Syst Man Cyb. 1989;19(5): 1264–1274. 10.1109/21.44046

[7] Galloway MM. Texture analysis using gray level run lengths. Comput Computer Graphics and Image Processing. 1975;4(2): 172–179. 10.1007/s00774-004-0536-9

[8] Thibault G, Fertil B, Navarro C, Pereira S, Cau P, Levy N, et al Texture Indexes and Gray Level Size Zone Matrix. Application to Cell Nuclei Classification. PRIP. 2009: 140–145.

[9] Yip S, Aerts H. Applications and limitations of radiomics. Phys Med Biol. 2016;61: R150–166. 10.1088/0031-9155/61/13/R150 27269645 PMC4927328

[10] Lemarignier C, Di Fiore F, Marre C, Hapdey S, Modzelewski R, Gouel P, et al Pretreatment Metabolic Tumour Volume Is Predictive of Disease-free Survival and Overall Survival in Patients With Oesophageal Squamous Cell Carcinoma. Eur J Nucl Med Mol Imaging. 2014;41: 2008–2016. 10.1007/s00259-014-2839-y 25037871

[11] Blom R, Steenbakkers IR, Lammering G, Vliegen R, Belgers EJ, De Jonge C, et al PET/CT-based metabolic Tumour Volume for Response Prediction of Neoadjuvant Chemoradiotherapy in Oesophageal Carcinoma. Eur J Nucl Med Mol Imaging. 2013;40: 1500–1506. 10.1007/s00259-013-2468-x 23764889

[12] Palie O, Michel P, Ménard J-F, Rousseau C, Rio E, Bridji B, et al The Predictive Value of Treatment Response Using FDG PET Performed on Day 21 of Chemoradiotherapy in Patients With Oesophageal Squamous Cell Carcinoma. A Prospective, Multicentre Study (RTEP3). Eur J Nucl Med Mol Imaging. 2013;40: 1345–1355. 10.1007/s00259-013-2450-7 23715903

[13] Hatt M, Visvikis D, Pradier O, Cheze-Le Rest C. Baseline 18F-FDG PET Image-derived Parameters for Therapy Response Prediction in Oesophageal Cancer. Eur J Nucl Med Mol Imaging. 2011; 38: 1595–1596. 10.1007/s00259-011-1834-9 21559979 PMC3375481

[14] Hatt M, Visvikis D, Albarghach NM, Tixier F, Pradier O, Cheze-Le Rest C. Prognostic Value of 18 F-FDG PET Image-based Parameters in Oesophageal Cancer and Impact of Tumour Delineation Methodology. Eur J Nucl Med Mol Imaging. 2011;38: 1191–1202. 10.1007/s00259-011-1755-7 21365252

[15] Hatt M, Majdoub M, Vallieres M, Tixier F, Cheze-Le Rest C, Groheux D, et al 18F-FDG PET Uptake Characterization Through Texture Analysis: Investigating the Complementary Nature of Heterogeneity and Functional Tumor Volume in a multi-Cancer Site Patient Cohort. J Nucl Med. 2015;56: 38–44. 10.2967/jnumed.114.144055 25500829

[16] Hyun SH, Choi JY, Shim YM, Kim K, Lee SJ, Cho YS, et al Prognostic Value of Metabolic Tumor Volume Measured by 18F-fluorodeoxyglucose Positron Emission Tomography in Patients With Esophageal Carcinoma. Ann Surg Oncol. 2010;17: 115–122. 10.1245/s10434-009-0719-7 19826877

[17] Tixier F, Cheze-le Rest C, Hatt M, Albarghach NM, Pradier O, Metges JP, et al Intratumor Heterogeneity Characterized by Textural Features on Baseline 18F-FDG PET Images Predicts Response to Concomitant Radiochemotherapy in Esophageal Cancer. J Nucl Med. 2011;52: 369–378. 10.2967/jnumed.110.082404 21321270 PMC3789272

[18] Parmar C, Grossmann P, Bussink J, Lambin P, Aerts H. Machine Learning methods for Quantitative Radiomic Biomarkers. Sci Rep. 2015;5: 13087 10.1038/srep13087 26278466 PMC4538374

[19] Raman SP, Chen Y, Schroeder JL, Huang P, Fishman EK. CT Texture Analysis of Renal Masses: Pilot Study Using Random Forest Classification for Prediction of Pathology. Acad Radiol; 2014;21(12): 1587–1596. 10.1016/j.acra.2014.07.023 25239842 PMC4352301

[20] Brooks FJ and Grigsby PW. The Effect of Small Tumor Volumes on Studies of Intratumoral Heterogeneity of Tracer Uptake. J Nucl Med. 2014;55(1): 37–42. 10.2967/jnumed.112.116715 24263086 PMC4017737

[21] Hatt M, Groheux D, Martineau A, Espié M, Hindié E, Giacchetti S, et al Comparison between 18F-FDG PET image-derived indices for early prediction of response to neoadjuvant chemotherapy in breast cancer. J Nucl Med. 2013;54(3): 341–349. 10.2967/jnumed.112.108837 23327900

[22] Orlhac F, Soussan M, Maisonobe JA, Garcia CA, Vanderlinden B, Buvat I. Tumor Texture Analysis in 18F-FDG PET: Relationships between Texture Parameters, Histogram Indices, Standardized Uptake Values, Metabolic Volumes, and Total Lesion Glycolysis. J Nucl Med. 2014;55: 414–422. 10.2967/jnumed.113.129858 24549286

[23] Herskovic A, Martz K, Al-Sarraf M, Leichman L, Brindle J, Vaitkevicius V, et al Combined Chemotherapy and Radiotherapy Compared with Radiotherapy Alone in Patients With Cancer of the Esophagus. N Engl J Med. 1992;326: 1593–1598. 10.1056/NEJM199206113262403 1584260

[24] Vauclin S, Doyeux K, Hapdey S, Edet-Sanson A, Vera P, Gardin I. Development of a generic thresholding algorithm for the delineation of 18F-FDG-PET- positive tissue: Application to the comparison of three thresholding models. Phys Med Biol. 2009;54: 6901–6916. 10.1088/0031-9155/54/22/010 19864698

[25] Hofheinz F, Lougovski A, Zöphel K, Hentschel M, Steffen IG, Apostolova I, et al Increased Evidence for the Prognostic Value of Primary Tumor Asphericity in Pretherapeutic FDG PET for Risk Stratification in Patients With Head and Neck Cancer. Eur J Nucl Med Mol Imaging. 2014;42: 429–437. 10.1007/s00259-014-2953-x 25416633

[26] Leijenaar RTH, Carvalho S, Velazquez ER, van Elmpt WJC, Parmar C, Hoekstra OS, et al Stability of FDG-PET Radiomics features: an integrated analysis of test-retest and inter-observer variability. Acta Oncol. 2013;52(7): 1391–1397. 10.3109/0284186X.2013.812798 24047337 PMC4533992

[27] Leijenaar RTH, Nalbantov G, Carvalho S, van Elmpt WJC, Troost EGC Boellaard R, et al The effect of SUV discretization in quantitative FDG-PET Radiomics: the need for standardized methodology in tumor texture analysis. Sci Rep. 2015;5: 11075 10.1038/srep11075 26242464 PMC4525145

[28] Breiman L. Random Forests. Mach Learn, 2001;45(1): 5–32.

[29] Yan J, Chu-Shern JL, Loi HY, Khor LK, Sinha AK, Quek ST, et al Impact of Image Reconstruction Settings on Texture Features in 18F-FDG PET. J Nucl Med. 2015;56(11): 1667–1673. 10.2967/jnumed.115.156927 26229145

[30] Mi H, Petitjean C, Dubray B, Vera P, Ruan S. Robust feature selection to predict tumor treatment outcome. Artif Intell Med. 2015;4(3): 195–204. 10.1016/j.artmed.2015.07.002 26303106

[31] Hand DJ, Till RJ. A Simple Generalisation of the Area Under the ROC Curve for Multiple Class Classification Problems. Mach Learn. 2001;45(2): 171–186.

[32] Chalkidou A, O’Doherty MJ, Marsden PK. False discovery rates in PET and CT Studies with texture feature: A systematic review. PLoS One. 2015;10(5): e0124165 10.1371/journal.pone.0124165 25938522 PMC4418696

[33] Hochberg Y, Benjamini Y. More Powerful Procedures for multiple significance testing. Stat Med. 1990;9(7): 811–818. 10.1002/sim.4780090710 2218183

[34] Altman DG, Lyman GH. Methodological Challenges in the Evaluation of Prognostic Factors in Breast Cancer. Breast Cancer Res Treat. 1998;52(1-3): 289–303. 10.1023/A:1006193704132 10066088

[35] Demsar J. Statistical Comparisons of Classifiers over Multiple Data Sets. JMLR. 2006;7: 1–30.

[36] Tan S, Kligerman S, Chen W, Lu M, Kim G, Feigenberg S, et al Spatial-temporal [18F]FDG-PET features for predicting pathologic response of esophageal cancer to neoadjuvant chemoradiation therapy. Int J Radiat Oncol Biol Phys. 2013;85(5): 1375–1382. 10.1016/j.ijrobp.2012.10.017 23219566 PMC3606641

[37] Tan S, Zhang H, Zhang Y, Chen W, D’Souza WD, Lu W. Predicting pathologic tumor response to chemoradiotherapy with histogram distances characterizing longitudinal changes in 18F-FDG uptake patterns. Med Phys. 2013;40(10): 101707 10.1118/1.4820445 24089897 PMC3785537

[38] Zhang H, Tan S, Chen W, Kligerman S, Kim G, D’Souza WD, et al Modeling pathologic response of esophageal cancer to chemoradiation therapy using spatial-temporal 18F-FDG PET features clinical parameters, and demographics. Int J Radiat Oncol Biol Phys. 2014;88(1): 195–203. 10.1016/j.ijrobp.2013.09.037 24189128 PMC3875172

[39] Giorgetti A, Pallabazzer G, Ripoli A, Solito B, Genovesi D, Lencioni M, et al Prognostic Significance of 2-Deoxy-2-[18F]-Fluoro-D-Glucose PET/CT in Patients With Locally Advanced Esophageal Cancer Undergoing Neoadjuvant Chemoradiotherapy Before Surgery: A Nonparametric Approach. Medicine (Baltimore). 2016;95(13): e3151 10.1097/MD.0000000000003151 27043676 PMC4998537

[40] Van Rossum PSN, Fried DV, Zhang L, Hofstetter WL, van Vulpen M, Meijer GJ, et al The Incremental Value of Subjective and Quantitative Assessment of 18F-FDG PET for the Prediction of Pathologic Complete Response to Preoperative Chemoradiotherapy in Esophageal Cancer. J Nucl Med. 2016;57(5): 691–700. 10.2967/jnumed.115.163766 26795288

[41] Ganeshan B, Skogen K, Pressney I, Coutroubis D, Miles K. Tumour heterogeneity in oesophageal cancer assessed by CT texture analysis: Preliminary evidence of an association with tumour metabolism, stage, and survival. Clin Radiol. 2012;67(2): 157–164. 10.1016/j.crad.2011.08.012 21943720

[42] Giganti F, Antunes S, Salerno A, Ambros A, Marra P, Nicoletti R, et al Gastric cancer: texture analysis from multidetector computed tomography as a potential preoperative prognostic biomarker. Eur Radiol. 2016; 10.1007/s00330-016-4540-y 27553932

[43] Giganti F, Salerno A, Ambrosi A, Chiari D, Orsenigo E, Esposito A, et al Prognostic utility of diffusionweighted MRI in oesophageal cancer: is apparent diffusion coefficient a potential marker of tumour aggressiveness? Radiol Med. 2016;121(3): 173–180. 10.1007/s11547-015-0585-2 26392393

[44] Desseroit MC, Visvikis D, Tixier F, Majdoub M, Perdrisot R, Guillevin R, et al Development of a nomogram combining clinical staging with 18F-FDG PET/CT image features in non-small-cell lung cancer stage I–III. Eur J Nucl Med Mol Imaging. 2016; 43(8): 1477–1485. 10.1007/s00259-016-3325-5 26896298 PMC5409954

[45] Gao X, Chu C, Li Y, Lu P, Wang W, Liu W, et al The method and efficacy of support vector machine classifiers based on texture features and multi-resolution histogram from 18F-FDG PET-CT images for the evaluation of mediastinal lymph nodes in patients with lung cancer. Eur J Radiol. 2015;84(2): 312–317. 10.1016/j.ejrad.2014.11.006 25487819

[46] Lartizien C, Rogez M, Niaf E, Ricard F. Computer-aided staging of lymphoma patients with FDG PET/CT imaging based on textural information. IEEE J Biomed Health Inform. 2014;18(3): 946–955. 10.1109/JBHI.2013.2283658 24081876

[47] Vallières M, Freeman CR, Skamene SR, El Naqa I. A radiomics model from joint FDG-PET and MRI texture features for the prediction of lung metastases in soft-tissue sarcomas of the extremities. Phys Med Biol. 2015;60(14): 5471–5496. 10.1088/0031-9155/60/14/5471 26119045

[48] Aerts HJ, Velazquez ER, Leijenaar RT, Parmar C, Grossmann P, Cavalho S, et al Decoding tumour phenotype by noninvasive imaging using a quantitative radiomics approach. Nat Commun. 2014;5: 4006 10.1038/ncomms5006 24892406 PMC4059926

[49] Tixier F, Hatt M, Cheze-Le Rest C, Le Pogam A, Corcos L, Visvikis Dimitris. Reproducibility of Tumor Uptake Heterogeneity Characterization Through Textural Feature Analysis in 18F-FDG PET. J Nucl Med. 2012;53(5): 693–700. 10.2967/jnumed.111.099127 22454484 PMC3779464

[50] Yu CH. Resampling methods: concepts, applications, and justification. PARE. 2003;8(19).

[51] Desbordes P, Modzelewski R, Ruan S, Pineau P, Vauclin S, Vera P, et al Feature selection for outcome prediction in oesophageal cancer using genetic algorithm and random forest classifier. Comput Med Imaging Graph. 2016; 10.1016/j.compmedimag.2016.12.002 28087102

[52] Orlhac F, Soussan M, Chouahnia K, Martinod E, Buvat I. 18F-FDG PET-Derived Textural Indices Reflect Tissue-Specific Uptake Pattern in Non-Small Cell Lung Cancer. PLoS One. 2015;10(12): e0145063 10.1371/journal.pone.0145063 26669541 PMC4682929

