# Supplementary material for: Predictive value of initial FDG-PET features for treatment response and survival in esophageal cancer patients treated with chemo-radiation therapy using a random forest classifier
Source: PLoS One. 2017 Mar 10;12(3):e0173208. doi: 10.1371/journal.pone.0173208 (PMC5345816; doi:10.1371/journal.pone.0173208)
Supplement: S1 File — Mean, standard diviation (SD), median, 1st and 3rd quartile (Q1, Q3) of absolute PET texture features (Table A). Parameters of the RF (Table B). Results of RF classification obtained with two different resampling methods (Table C). Groups of correlated features created with an absolute threshold value of the Spearman’s correlation coefficient varying from 0.7 to 0.9. The feature selected to represent each group for the next step is in bold (Table D). (PDF) [file pone.0173208.s001.pdf]

# Supporting Information

**Table A . Mean, standard diviation (SD), median,, 1st and 3rd quartile (Q1, Q3) of absolute PET texture features.**

| Feature | Mean | SD | Q1 | Median | Q3 |
|---|---|---|---|---|---|
| Energy (GLCM) | 0.04 | 0.07 | 0.01 | 0.02 | 0.04 |
| Entropy (GLCM) | 1.98 | 0.49 | 1.71 | 2.06 | 2.28 |
| Dissimilarity (GLCM) | 2.46 | 1.42 | 1.47 | 2.28 | 3.09 |
| Contrast (GLCM) | 14.46 | 16.11 | 4.89 | 9.44 | 17.34 |
| Homogeneity (GLCM) | 0.49 | 0.14 | 0.39 | 0.46 | 0.55 |
| IDM (GLCM) | 0.42 | 0.16 | 0.31 | 0.40 | 0.50 |
| Variance (GLCM) | 48.59 | 51.42 | 14.79 | 33.67 | 66.36 |
| Cluster shade (GLCM) | 529.86 | 826.77 | 72.46 | 202.62 | 582.65 |
| Cluster tendency (GLCM) | 31573 | 63406 | 1749 | 7159 | 35112 |
| Correlation (GLCM) | 0.66 | 0.15 | 0.59 | 0.69 | 0.77 |
| Coarseness (GLDM) | 0.01 | 0.01 | 0.01 | 0.01 | 0.02 |
| Contrast (GLDM) | 0.15 | 0.11 | 0.08 | 0.13 | 0.18 |
| Busyness (GLDM) | $1.69E^{+12}$ | $2.08E^{+12}$ | $6.05E^{+11}$ | $1.02E+^{+12}$ | $1.76E^{+12}$ |
| Complexity (GLDM) | 451028 | 915384 | 36329 | 102725 | 315937 |
| Strength (GLDM) | 2.26 | 2.65 | 0.79 | 1.54 | 2.50 |
| SZE (GLSZM) | 0.41 | 0.15 | 0.35 | 0.42 | 0.50 |
| LZE (GLSZM) | 12830 | 41623 | 99 | 577 | 3029 |
| LGZE (GLSZM) | 0.08 | 0.10 | 0.03 | 0.04 | 0.07 |
| HGZE (GLSZM) | 106.65 | 81.27 | 55.47 | 90.00 | 125.10 |
| SZLGE (GLSZM) | 0.02 | 0.02 | 0.01 | 0.01 | 0.02 |
| SZHGE (GLSZM) | 56.36 | 58.77 | 21.94 | 37.58 | 61.64 |
| LZLGE (GLSZM) | 3522.03 | 12307.38 | 9.17 | 66.45 | 422.18 |
| LZHGE (GLSZM) | 103787 | 334984 | 3969 | 11267 | 38057 |
| GLNUz (GLSZM) | 12.66 | 11.89 | 5.49 | 8.84 | 15.97 |
| ZLNU (GLSZM) | 48.62 | 62.55 | 7.65 | 24.33 | 59.52 |
| ZP (GLSZM) | 0.16 | 0.14 | 0.05 | 0.13 | 0.22 |

## A Determination of the RF optimal parameters

The influence of several parameters of the proposed feature selection method has been evaluated: the resampling method, the threshold value of the Spearman's correlation coefficient, and $T$ the number of trees of the RF.

Table B shows the different parameters of the RF that were evaluated. The performances studied were the area under the curve of the ROC analysis and the error of classification (%). Because of the small number of observations in the database, the evaluation protocol was done using random permutations. As explained in the article, this process randomly divides the database into 2 subsets: two-thirds of the data are used for the training sample and one-third for the test sample. This process is repeated 10 times, leading to average and standard deviation of performance indices. Performances were compared using the Wilcoxon signed-rank test [35] with an $\alpha$ risk of 5%.

**Table B . Parameters of the RF.**

| Parameters | Values |
|---|---|
| Resampling method | Absolute or relative |
| Threshold of the Spearman's coefficient | 0.7, 0.8 and 0.9 |
| Number $T$ of trees of the RF | 100 to 500 (step of 50) |

## B Influence of the resampling method

Two main methods have been proposed in the literature to resample FDG-PET images. The first is a relative gray-level resampling where each tumor is resampled with $B$, a number of gray levels set by the user according to [22] and [52]:

$$R_{\text{rel}}(i) = \text{round}\left(B \times \frac{SUV(i) - \text{SUV}_{\text{min}}}{\text{SUV}_{\text{max}} \text{ - } \text{SUV}_{\text{min}}}\right) \quad (1)$$

where $SUV(i)$ is the initial SUV of voxel $i$, $R_{\text{rel}}(i)$ is the new intensity after the relative resampling process. $\text{SUV}_{\text{min}}$ and $\text{SUV}_{\text{max}}$ are the minimum and the maximum intensity of the studied tumor, respectively. Thus, each tumor has its own number $B$ of gray levels, set to 64.

The second is an absolute linear gray-level resampling according to [26] and [27]:

$$R_{\text{abs}}(i) = \text{round}(D \times SUV(i)) \quad (2)$$

where $SUV(i)$ is the initial SUV of voxel $i$ and $R_{\text{abs}}(i)$ is the new intensity after the absolute resampling process based on $D$ the intensity step D set to 0.5.

Texture features were extracted 2 times according to these 2 methods. Table C shows the results of the RF classifications obtained with these 2 sets of features.

**Table C . Results of RF classification obtained with two different resampling methods.**

| Study | Resampling | $\text{RF}_{\text{err}}$ (%) | AUC | Se (%) | Sp (%) | $p$-value Wilcoxon |
|---|---|---|---|---|---|---|
| | | | | | | signed rank test |
| Predictive | Relative | 35±12 | 0.675±0.119 | 64±24 | 78±25 | 0.04 |
| | Absolute | 21±9 | 0.836±0.105 | 82±9 | 91±12 | |
| Pronostique | Relative | 39±9 | 0.560±0.110 | 66±22 | 63±23 | 0.01 |
| | Absolute | 28±5 | 0.822±0.059 | 69±9 | 95±6 | |

The Wilcoxon signed-rank test revealed that absolute resampling gives significantly better results than relative resampling in our database.

## C Influence of the threshold value of the Spearman's correlation coefficient

Table D shows the different correlation groups obtained with 3 different threshold values ($|\rho| = 0.7$, 0.8, or 0.9). Furthermore, results of the classification after feature selection are shown in S1 Fig. The Wilcoxon signed-rank test did not show a significant difference.

**S1 Fig. Results of the RF classification according to the absolute threshold value of the Spearman's correlation coefficient (a) for the predictive study and (b) for the prognostic study.**

**Table D . Groups of correlated features created with an absolute threshold value of the Spearman's correlation coefficient varying from 0.7 to 0.9. The feature selected to represent each group for the next step is in bold.**

| $\rho$ | Grp | Features |
|---|---|---|
| 0.7 | 1 | **Patient's usual weight** - Patient's current weight |
| | 2 | **NRI** - Albumin level - Malnutrition |
| | 3 | $\mathbf{V_{10}}$**-**$\mathbf{V_{90}}$ - $V_{90}$ |
| | 4 | **ZLNU** - Cluster Shade (GLCM) - SZE |
| | 5 | **Energy** - Entropy - Kurtosis - Skewness |
| | 6 | **MTV** - TLG - $_{sum}$SUV - Correlation (GLCM) - Coarseness (GLDM) - Busyness (GLDM) - GLNUz |
| | 7 | $\mathbf{SUV_{max}}$ - $SUV_{10}$ - Variance (GLCM) - HGZE - Cluster tendency (GLCM) - $SUV_{mean}$ - $SUV_{peak}$ |
| | | - SZHGE - SD - Complexity (GLDM) - $SUV_{10}$-$SUV_{90}$ - LGZE - Entropy (GLCM) - Contrast (GLCM) |
| | | - Dissimilarity (GLCM) - ZP - Strength (GLDM) - $SUV_{90}$ |
| | 8 | **Homogeneity (GLCM)** - IDM (GLCM) - Contrast (GLDM) - Energy (GLCM) - LZE - LZHGE |
| | | - LZLGE |
| | Indpt | **11 clinical features - $\mathbf{V_{10}}$ - COV - Sphericity - SZLGE** |
| 0.8 | 4 | **ZLNU** - Cluster Shade (GLCM) |
| | 5 | **Energy** - Entropy |
| | 6 | **MTV** - TLG - $_{sum}$SUV - Correlation (GLCM) |
| | 7 | $\mathbf{SUV_{max}}$ - $SUV_{10}$ - Variance (GLCM) - HGZE - Cluster tendency (GLCM) - $SUV_{mean}$ - $SUV_{peak}$ |
| | | - SZHGE - SD - Complexity (GLDM) - $SUV_{10}$-$SUV_{90}$ - LGZE |
| | 8 | **Homogeneity (GLCM)** - IDM (GLCM) - Contrast (GLDM) - Energy (GLCM) - LZE - LZHGE |
| | | - LZLGE - Dissimilarity (GLCM) - Contrast (GLCM) - ZP - Entropy (GLCM) - Strength (GLDM) |
| | 9 | **Busyness (GLDM)** - Coarseness (GLDM) - Sphericity |
| | Indpt | **11 clinical features - $\mathbf{V_{10}}$ - $\mathbf{SUV_{90}}$ - COV - Kurtosis - Skewness - SZE - SZLGE - GLNUz** |
| 0.9 | 4 | **ZLNU** - Cluster Shade (GLCM) |
| | 5 | **Energy** - Entropy |
| | 6 | **MTV** - TLG - $_{sum}$SUV |
| | 7 | $\mathbf{SUV_{max}}$ - $SUV_{10}$ - Variance (GLCM) - HGZE - Cluster tendency (GLCM) - $SUV_{mean}$ - $SUV_{peak}$ |
| | | - SD - SZHGE |
| | 8 | **Homogeneity (GLCM)** - IDM (GLCM) - Contrast (GLDM) - Dissimilarity (GLCM) |
| | | - Contrast (GLCM) - ZP - Entropy (GLCM) |
| | 9 | **Busyness (GLDM)** - Coarseness (GLDM) - Sphericity |
| | 10 | **LZE** - LZHGE - LZLGE |
| | Indpt | **11 clinical features - COV - Skewness - Kurtosis - $\mathbf{SUV_{90}}$ - $\mathbf{SUV_{10}}$-$\mathbf{SUV_{90}}$ - $\mathbf{V_{10}}$** |
| | | **- Energy (GLCM) - Correlation (GLCM) - SZE - LGZE - SZLGE - GLNUz** |
| | | **- Complexity (GLDM) - Strength (GLDM)** |

## D Influence of $T$ the number of trees of the RF

The influence of the number of trees of the RF was evaluated by varying $T$ from 50 to 500. Results of the classification are shown in S2 Fig. The Wilcoxon signed-rank test did not show a significant difference.

**S2 Fig. Results of the RF classification according to $T$ the number of trees of the RF (a) for the predictive study and (b) for the prognostic study.**
