# Supplementary figures and images for: Predictive value of initial FDG-PET features for treatment response and survival in esophageal cancer patients treated with chemo-radiation therapy using a random forest classifier
Source: PLoS One. 2017 Mar 10;12(3):e0173208. doi: 10.1371/journal.pone.0173208 (PMC5345816; doi:10.1371/journal.pone.0173208)

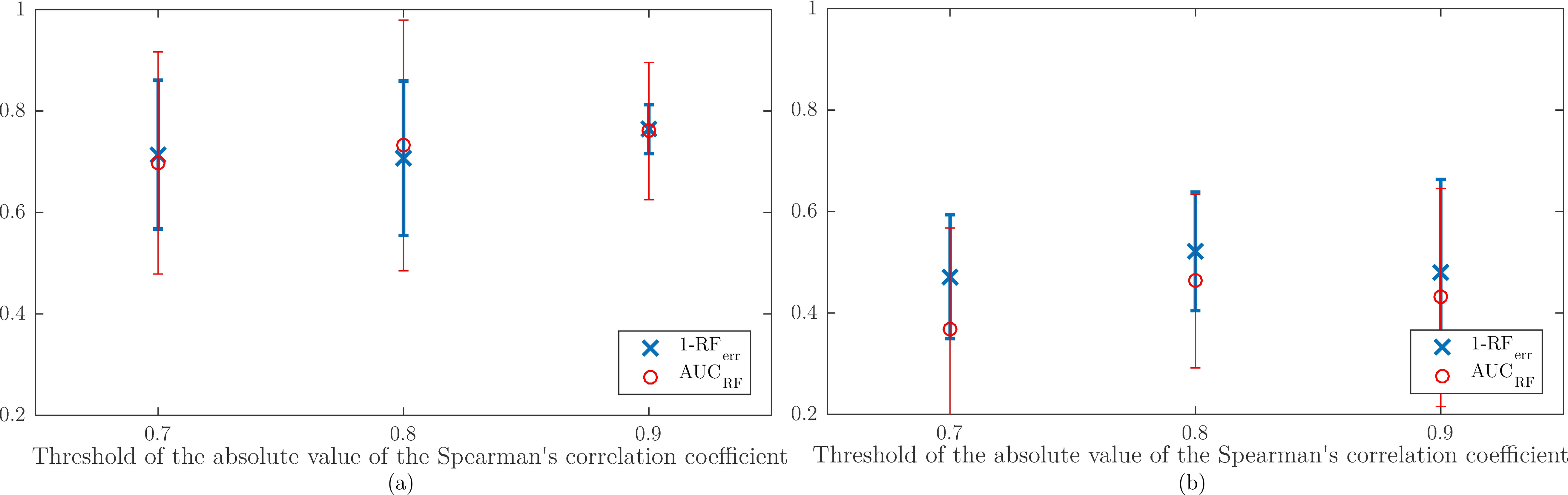

Supplement: S1 Fig — (TIF) [file pone.0173208.s002.tif]

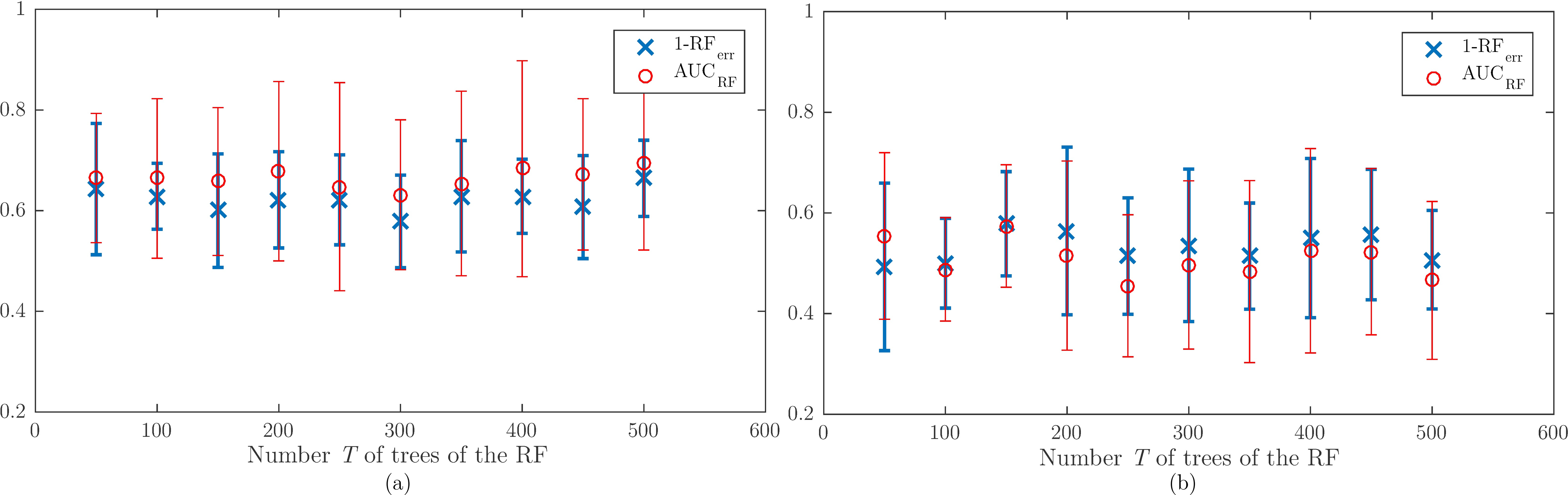

Supplement: S2 Fig — (TIF) [file pone.0173208.s003.tif]
